# Rescue Vedolizumab Therapy for a Rare Case of Complicated Severe Ulcerative Colitis: A Case Report and Literature Review

**DOI:** 10.3390/jcm15135166

**Published:** 2026-07-02

**Authors:** Shih-Tsung Fu, Kai-Po Chang, Wei-Jhe Hong, Jen-Wei Chou, Yi-Hua Wu

**Affiliations:** 1 Center for Digestive Medicine, Department of Internal Medicine, China Medical University Hospital, Taichung 404, Taiwan; 038188@too.caaumed.org.tw (S.-T.F.); u111305801@cmu.edu.tw (J.-W.C.); 2 School of Medicine, College of Medicine, China Medical University, Taichung 404, Taiwan; 017179@tool.caaumed.org.tw (K.-P.C.); 012710@tool.caaumed.org.tw (W.-J.H.); 3 Department of Pathology, China Medical University Hospital, Taichung 404, Taiwan; 4 Division of Rheumatology and Immunology, Department of Internal Medicine, China Medical University Hospital, Taichung 404, Taiwan

**Keywords:** ulcerative colitis, primary sclerosing cholangitis, systemic lupus erythematosus, vedolizumab, autoimmune overlap, inflammatory bowel disease

## Abstract

**Background:** Ulcerative colitis (UC) is a chronic inflammatory bowel disease associated with extraintestinal manifestations, including primary sclerosing cholangitis (PSC). Systemic lupus erythematosus (SLE) is a multisystem autoimmune disease that rarely coexists with UC or PSC. The concurrent occurrence of UC, PSC, and SLE in a single individual represents a unique diagnostic and therapeutic challenge. Vedolizumab, a gut-selective biologic agent, is effective for managing UC; however, its utility in patients presenting with this triad of conditions has not yet been explored. **Case summary:** A 32-year-old man presented with a 10-year history of recurrent upper abdominal pain, frequently accompanied by high-grade fever, along with recent onset of jaundice, diarrhea, hematochezia, and chronic rashes. Diagnostic evaluation confirmed PSC, SLE, and severe UC. During hospitalization, the patient also developed bacteremia. Initial management of UC with mesalazine and immunosuppressants (azathioprine followed by cyclosporine) resulted in limited clinical improvement. Vedolizumab was subsequently initiated, resulting in marked clinical improvements and near-complete endoscopic remission of UC. PSC and SLE remained clinically stable with ongoing therapies; however, the patient is currently awaiting liver transplantation for PSC. **Conclusions:** This case highlights the potential utility of vedolizumab in the treatment of UC in patients with concurrent PSC and SLE.

## 1. Introduction

Ulcerative colitis (UC) is an inflammatory bowel disease (IBD) characterized by diffuse, continuous mucosal inflammation predominantly affecting the colon. It is commonly associated with symptoms such as bloody diarrhea, abdominal pain, and urgency. Extraintestinal manifestations, including articular, cutaneous, and ocular manifestations, as well as primary sclerosing cholangitis (PSC), occur in approximately 7–17% of patients with UC [1]. PSC and UC exhibit significant overlap. IBD is present in 70–80% of Northern Europeans and 20–50% of Asians with PSC, most commonly in the form of UC [2]. The development of PSC in patients with IBD is associated with a higher mortality risk compared with those with IBD alone, with a reported hazard ratio of 3.20 [3,4]. Furthermore, the coexistence of PSC and IBD increases the risk of complications such as colorectal cancer and end-stage liver disease, often necessitating liver transplantation.

Systemic lupus erythematosus (SLE) is an autoimmune disease characterized by multisystem and multiorgan involvement, driven by the production of autoantibodies targeting nuclear antigens. Gastrointestinal manifestations occur in approximately 50% of patients with SLE [5], with lupus enteritis reported in 3.4–5.8% of patients with SLE in Asia [6,7,8]. However, SLE rarely overlaps with UC or PSC. Approximately 0.4% of patients with SLE have UC, while SLE is reported in about 1.7% of patients with PSC [5]. The concurrent occurrence of UC, PSC, and SLE in a single individual is exceptionally rare and presents unique diagnostic and therapeutic challenges.

Although the coexistence of UC, PSC, and SLE has been reported, therapeutic approaches and outcomes have varied considerably [9,10,11]. For instance, one patient underwent intestinal resection for UC before the diagnoses of PSC and SLE were established [9], whereas another study described a case of SLE that was refractory to combination therapy [10]. These reports suggest that management may not be universally effective in the setting of this complex triple autoimmune burden.

Over the past two decades, the treatment of moderate-to-severe UC has shifted toward biologic therapies [12]. In this context, early recognition of overlapping autoimmune conditions is increasingly important to avoid unnecessary surgical interventions and to guide the selection of the most appropriate biologic agent. Recent advances in ulcerative colitis research have expanded the understanding of disease pathogenesis and therapeutic targeting. In addition to dysregulated adaptive immune responses, increasing evidence highlights the roles of epithelial barrier dysfunction, intestinal microbiome alterations, and aberrant innate immune activation in driving chronic intestinal inflammation. These advances have facilitated the development of novel therapeutic approaches, including selective Janus kinase inhibitors, sphingosine-1-phosphate receptor modulators, and precision biologic therapies targeting specific inflammatory pathways. Despite these developments, optimal treatment selection remains challenging in patients with complex autoimmune overlap syndromes, particularly when concomitant conditions increase the risk associated with systemic immunosuppression. Therefore, individualized treatment strategies remain essential in this unique patient population [13,14,15].

Vedolizumab, a gut-selective α4β7 integrin antagonist, has demonstrated efficacy in inducing and maintaining remission in patients with moderate-to-severe UC by inhibiting the trafficking of gut-homing T cells, thereby reducing intestinal inflammation [13]. However, the efficacy of vedolizumab in patients with concurrent PSC, SLE, and UC has not been investigated. Therefore, this report describes a patient who achieved significant clinical and endoscopic remission of UC following vedolizumab therapy, while SLE and PSC remained well controlled with ongoing management.

## 2. Case Presentation

A 32-year-old man of Southern Min ethnicity, employed as a supermarket clerk, presented to our outpatient department with a 10-year history of upper abdominal pain. He described the pain as colicky, intermittent, and predominantly postprandial, particularly after consuming oily or spicy foods. The discomfort was localized to the upper abdomen, occasionally radiating to the back and right shoulder, with each episode lasting more than 30 min and having moderate intensity (numeric rating scale: 4–5/10). Approximately 10 years prior to presentation, he underwent a cholecystectomy at a local hospital. However, this surgery did not alleviate his pain, which he managed symptomatically over the ensuing years without extensive investigation. At the time of referral, he reported mild nausea, anorexia, diarrhea (>5 times/day), intermittent hematochezia for a month, poor appetite, weight loss exceeding 10 kg over 6 months (>10% of body weight), fatigue, jaundice, and generalized pruritus. The patient frequently experienced recurrent fever with body temperature exceeding 38 °C during pain episodes. He also reported a chronic, intensely pruritic rash primarily affecting the extremities, cheeks, and posterior auricles, which had been present since adolescence. However, he had never sought evaluation for this rash, which was not correlated with his gastrointestinal symptoms. He denied smoking, alcoholism, or illicit drug use, and he had no relevant travel or contact history. Family history was positive for gallstones but negative for malignancies or autoimmune disorders.

Physical examination revealed fatigue, jaundice, and icteric sclera. Abdominal examination demonstrated mild tenderness in the upper quadrants with hyperactive bowel sounds. Murphy’s sign was negative, and there were no signs of peritoneal irritation. Dermatological examination revealed a diffuse, scattered maculopapular skin rash over the extensor surfaces of the extremities. The patient was admitted for further evaluation, during which his vital signs remained within normal limits. Laboratory investigations demonstrated marked cholestasis with elevated total bilirubin (5.68 mg/dL), direct bilirubin (3.84 mg/dL), alkaline phosphatase (1120 IU/L), and gamma-glutamyl transferase (883 IU/L). Liver transaminases were also elevated, including aspartate aminotransferase (102 IU/L) and alanine aminotransferase (97 IU/L). Additional findings included mildly prolonged prothrombin time (12.7 s) and hypoalbuminemia (3.1 g/dL). Serological testing for viral hepatitis, including antigen and antibody assays, was negative. Inflammatory markers were elevated, including highly sensitive C-reactive protein (6.76 mg/dL) and erythrocyte sedimentation rate (87 mm/hour). Autoimmune screening demonstrated positivity for antinuclear antibody (1:80; fine speckled, homogenous pattern), anti-double-stranded DNA antibody (383.561 U/mL), anti-smooth muscle antibody, and perinuclear anti-neutrophil cytoplasmic antibody, along with elevated immunoglobulin G (3200 mg/dL). In contrast, anti-mitochondrial antibody was negative, and complement levels (C3 and C4) were within normal limits. Abdominal ultrasonography (Figure 1) and computed tomography confirmed intrahepatic biliary dilatation in the absence of obstructing calculi and demonstrated features suggestive of parenchymal liver disease with splenomegaly (Figure 2a,b). Endoscopic retrograde cholangiopancreatography (ERCP) demonstrated diffuse irregularities, multifocal strictures, and dilatation of the intrahepatic bile ducts (Figure 3a,b). Biliary sludge and small stones were removed, and an endoscopic retrograde biliary drainage stent was placed to facilitate bile drainage. Magnetic resonance cholangiopancreatography corroborated the ERCP findings, which were characteristic of PSC (Figure 4).

The patient was initially treated for acute cholangitis secondary to PSC with intravenous antibiotics and ursodeoxycholic acid. Concurrently, intravenous corticosteroid therapy (20 mg daily for 3 days) and hydroxychloroquine (200 mg daily) were initiated for SLE. Colonoscopy revealed continuous inflammation of the colonic mucosa, starting in the rectum and extending proximally, characterized by loss of vascular pattern, erosions, ulcerations, and spontaneous bleeding (Figure 5a,b). Histopathological examination demonstrated focal architectural distortion, goblet cell depletion, cryptitis, and basal plasmacytosis with a Nancy index of 4, consistent with a diagnosis of UC (Figure 6a,b).

Following improvement in gastrointestinal bleeding, oral prednisolone (10 mg daily) and mesalazine (1000 mg daily) were introduced for UC. After confirmation of severe ulcerative colitis (Mayo endoscopic score 3, E3 disease extent), azathioprine (50 mg orally every 3 days) was initiated. However, after one month of treatment, hepatotoxicity developed, prompting dose reduction to 25 mg every 3 days. Cyclosporine (50 mg daily) was subsequently added. Despite these interventions, intermittent hematochezia and recurrent elevations of liver enzymes persisted (Table 1).

During hospitalization, the patient developed acute cholangitis complicated by *Esche richia coli* bacteremia. Blood cultures yielded *E. coli*, which was considered secondary to biliary tract infection associated with PSC. The patient received intravenous flomoxef for 7 days, resulting in complete clinical resolution. Follow-up blood cultures were negative.

Given the persistent UC activity, azathioprine-related hepatotoxicity, recurrent cholangi tis, and increased infection risk associated with systemic immunosuppression, vedolizumab was selected as rescue therapy. Vedolizumab was administered intravenously at a dose of 300 mg at weeks 0, 2, and 6, followed by maintenance therapy every 8 weeks.

After one year of follow-up, stool frequency decreased from five bowel movements per day to one bowel movement per day, and hematochezia completely resolved. The total Mayo score improved from 11 to 1, while the Mayo endoscopic score improved from 3 to 1 (Figure 7a,b). Inflammatory markers also improved substantially, with C-reactive protein decreasing from 12 mg/L to 1 mg/L and erythrocyte sedimentation rate decreasing from 17 mm/h to 5 mm/h. Furthermore, fecal calprotectin levels declined from 256 μg/g to 12 μg/g. The patient remained clinically stable and was able to resume normal daily activities. During follow-up, PSC and SLE remained clinically stable, although the patient continues to await liver transplantation (Table 2).

## 3. Discussion

The concurrent presentation of PSC, UC, and SLE in this single patient represents an exceedingly rare confluence of autoimmune disorders, posing substantial diagnostic and therapeutic challenges. In this case, the diagnostic process was notably protracted, as the patient had a decade-long history of abdominal pain that was initially misattributed to postcholecystectomy syndrome. The definition of postcholecystectomy syndrome remains imprecise. In general, symptoms arising within 3 years of surgery are more likely to be nonbiliary in origin. However, those occurring beyond 3 years might be biliary-related, but they are mainly attributable to new pathological processes within the altered biliary system rather than to cholecystectomy itself [16]. Given that the patient’s abdominal pain had persisted for a decade since the preoperative period, the possibility of misdiagnosis was suspected [16,17]. Eventually, a comprehensive workup revealed PSC based on characteristic cholangiographic findings on ERCP/magnetic resonance cholangiopancreatography and cholestatic liver enzyme levels [18]. Furthermore, the presence of a chronic pruritic rash since adolescence, together with persistently elevated titers of anti-double-stranded DNA antibodies and positivity for antinuclear antibodies, fulfilled the European League Against Rheumatism/American College of Rheumatology classification criteria for SLE [19].

A key clinical challenge is differentiating severe pancolitis of UC from potential SLE-related gastroenteritis. Hematochezia, as observed in our patient, is characteristic of UC [20], whereas lupus enteritis often exhibits non-specific manifestations such as abdominal pain, nausea, vomiting, and diarrhea, associated with flares of other lupus symptoms [21]. In this case, the endoscopic findings of diffuse mucosal hyperemia and erosions throughout the entire colon, along with the histopathological features, namely chronic colitis with crypt architectural distortion, goblet cell depletion, cryptitis, and basal plasmacytosis, are classical hallmarks of UC rather than vasculitis or the less specific inflammatory changes typically observed in SLE-associated enteritis [21].

This case highlights the immunological rarity of the UC–PSC–SLE triad. The strong association between PSC and UC is well recognized and is thought to be mediated by the gut–liver axis, in which impaired bile acid homeostasis leads to gut microbiota dysbiosis and eventually results in hepatic inflammation [22]. Although SLE less frequently overlaps with PSC or UC, genome-wide association studies have identified correlations. A study employing Mendelian randomization suggested that PSC is linked to an increased risk of SLE (odds ratio = 1.33) but not vice versa, suggesting a unidirectional relationship [23]. Regarding UC and SLE, genetic overlap analyses revealed a significant genetic correlation between IBD (including UC) and SLE [24]. This genetic link is further supported by shared diagnostic markers, such as killer cell lectin-like receptor F1, granzyme K, killer cell lectin-like receptor B1, CD40 ligand, and interleukin 7 receptor, as well as similar immune cell infiltration patterns, including increased monocyte counts and decreased resting natural killer cell counts [25]. Although a direct pathophysiological link has not been fully established for some of these overlapping relationships, our present case underscores the importance of maintaining a broad differential diagnosis and conducting a comprehensive workup, particularly when diseases possibly share genetic susceptibility.

Several factors may explain the favorable intestinal response observed in this patient. First, vedolizumab selectively blocks α4β7 integrin-mediated lymphocyte trafficking to the gastrointestinal tract, thereby reducing intestinal inflammation while minimizing systemic immunosuppression. Second, recurrent cholangitis and Escherichia coli bacteremia increased concerns regarding infection risk associated with broader immunosuppressive agents. Therefore, vedolizumab was selected because its gut-selective mechanism offered a potentially safer therapeutic approach in this clinical setting. Third, successful control of biliary infection and maintenance of biliary drainage may also have contributed to the patient’s overall clinical improvement. Therefore, the favorable outcome observed in this case likely reflects the combined effects of vedolizumab and comprehensive multidisciplinary management rather than vedolizumab alone.

The introduction of vedolizumab marked a pivotal point in the management of severe UC in this patient. Systemically, vedolizumab has been associated with higher rates of clinical remission and fewer serious adverse events than tumor necrosis factor (TNF) antagonist therapy or placebo [26,27]. In patients with UC and Crohn’s disease, vedolizumab treatment was associated with lower overall infection rates and did not increase the risk of serious or opportunistic infections relative to placebo [27]. A meta-analysis of 11 cohort studies reported that vedolizumab was associated with a 32% lower risk of serious infections compared with TNF antagonists, especially in the UC population [28]. Importantly, vedolizumab did not increase the incidence of gastrointestinal infections despite its gut-targeted mechanism. In an integrated analysis of vedolizumab registration trials, therapy was associated with a numerically, but not statistically significant, higher number of enteric infections [29], suggesting no meaningful increase in infection risk. Moreover, vedolizumab appeared to confer protection against severe Clostridium difficile infection and cytomegalovirus colitis in patients with UC compared with the effects of anti-TNFα agents [30]. These findings support vedolizumab as a favorable therapeutic option for patients with UC who are at elevated risk of infection.

In our patient, vedolizumab achieved near-complete endoscopic remission of UC, despite the presence of polyautoimmunity. Given the shared pathophysiological mechanisms between PSC and UC, several studies have investigated the efficacy of vedolizumab for both conditions, but no significant improvements in liver biochemistry or the Mayo PSC Risk Score were recorded [31,32]. However, a subset of patients with more advanced cirrhosis demonstrated a 20% reduction in alkaline phosphatase levels [31]. Overall, the effectiveness of vedolizumab in patients with coincident UC and PSC appears comparable to that in patients with UC alone [31,32]. Mild liver-related adverse events, including hepatitis and transient elevations of liver enzymes, have been reported in only a small number of patients, representing a potential but uncommon concern [33]. Although it remains unclear whether vedolizumab predisposes patients with PSC to bile duct injury [34], current evidence indicates that hepatotoxicity rates are comparable in patients with UC with or without coexisting PSC [31]. Monitoring liver function during therapy is therefore recommended as a precaution [34], particularly in patients with underlying liver disease or a history of azathioprine-induced hepatotoxicity.

To date, no cases of UC, PSC, and SLE treated with vedolizumab have been reported. Only a single case of overlapping Crohn’s disease and SLE has been reported, in which clinical remission was achieved despite the need for surgical repair of a rectovaginal fistula [35]. Notably, drug-induced lupus (DIL), an autoimmune condition with features resembling SLE, has been reported in association with vedolizumab therapy, potentially complicating clinical management. Nevertheless, this phenomenon has been observed in only three cases [36,37,38], all of which occurred following failure of TNF-α antagonist therapy, a known cause of DIL [39], prior to initiating vedolizumab therapy. Differentiating DIL from an SLE exacerbation requires careful evaluation of drug exposure timelines, autoantibody profiles, and symptom resolution after withdrawal of the offending drug. Overall, vedolizumab appears to be an effective and generally safe treatment for UC, even in complex cases with polyautoimmunity, although careful monitoring for potential complications remains essential.

## 4. Limitations

Several limitations of this report should be acknowledged. First, this study describes a single patient; therefore, causal relationships between vedolizumab treatment and the observed clinical improvements cannot be definitively established. The favorable outcome may have been influenced by multiple factors, including prior immunosuppressive therapies, antibiotic treatment, biliary drainage procedures, and the natural fluctuation of disease activity.

Second, although significant improvements in clinical symptoms, inflammatory markers, fecal calprotectin levels, and endoscopic findings were observed, the follow-up duration was limited to one year. Longer-term follow-up is necessary to determine the durability of remission, long-term safety, and the potential impact of vedolizumab on disease progression.

Third, the improvement observed in this case primarily involved ulcerative colitis. PSC and SLE remained clinically stable during follow-up; however, objective evidence demonstrating a direct therapeutic effect of vedolizumab on these conditions was not identified. Therefore, the findings should not be interpreted as evidence supporting the efficacy of vedolizumab for the entire UC–PSC–SLE overlap syndrome.

Finally, because this combination of ulcerative colitis, primary sclerosing cholangitis, and systemic lupus erythematosus is exceptionally rare, the generalizability of our observations is inherently limited. Additional case reports, multicenter registries, and prospective studies are needed to better define the role of vedolizumab in patients with complex autoimmune overlap syndromes.

## 5. Conclusions

This case of a 32-year-old man with concurrent PSC, SLE, and UC demonstrated the successful application of vedolizumab in a complex clinical scenario. By sharing this uncommon case, we aim to increase awareness of UC complicated by PSC and SLE and highlight the significance of early detection to prevent severe consequences. Vedolizumab was associated with marked clinical and endoscopic improvement in this patient with severe UC concomitant with PSC and SLE. However, causality cannot be established from a single case report, and further studies are needed to clarify its safety and effectiveness in similar clinical settings.

## Figures and Tables

**Figure 1:**
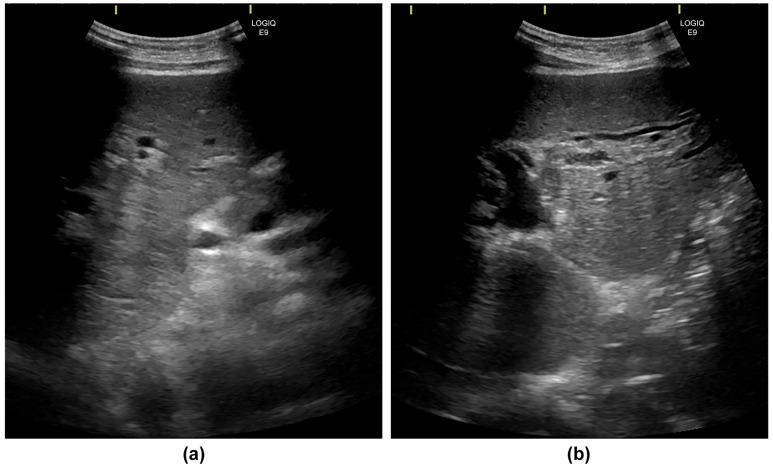
Abdominal ultrasound. (**a**) Parenchymal liver disease; (**b**) Intrahepatic biliary dilatation.

**Figure 2:**
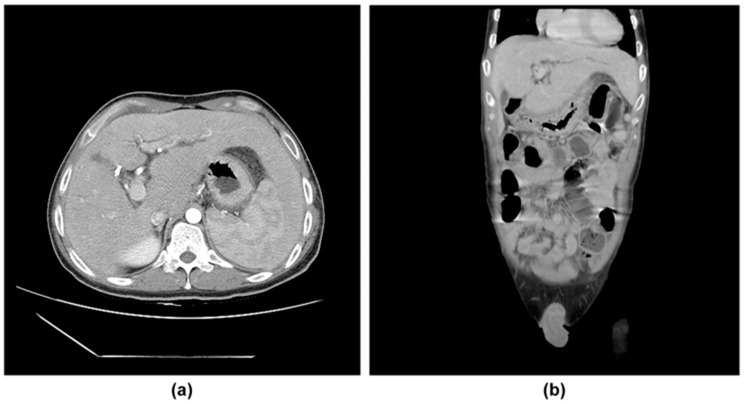
Computed tomography (CT) scan. (**a**) Parenchymal liver disease with splenomegaly; (**b**) Intrahepatic biliary dilatation.

**Figure 3:**
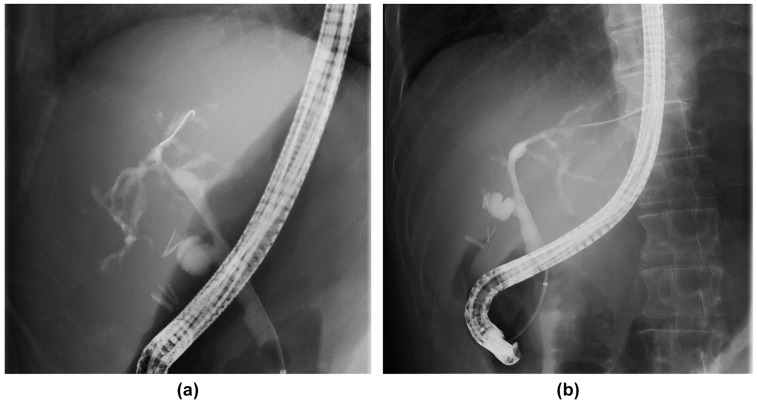
Endoscopic retrograde cholangiopancreatography (ERCP). (**a**) Irregularities, strictures, and dilatation of the right intrahepatic ducts; (**b**) Irregularities, strictures, and dilatation of the left intrahepatic ducts.

**Figure 4:**
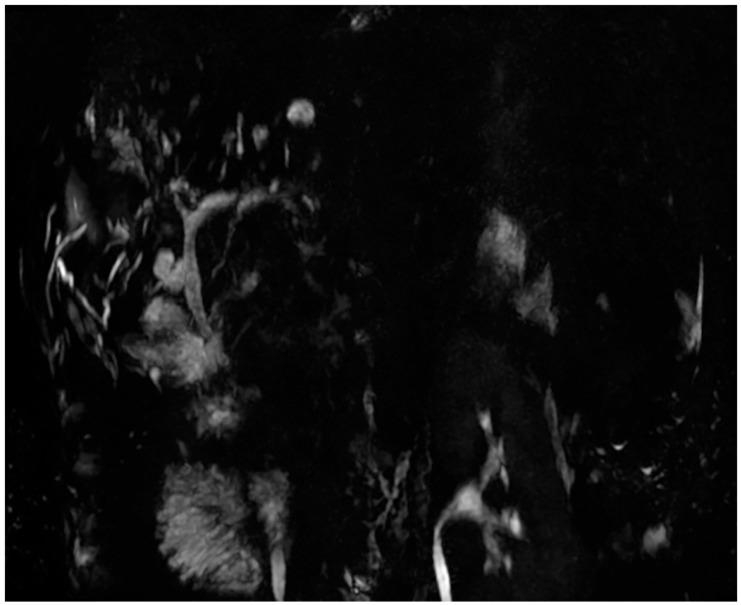
Magnetic resonance cholangiopancreatography (MRCP). Characteristic primary sclerosing cholangitis: Irregularities, strictures, and dilatation of biliary tracts.

**Figure 5:**
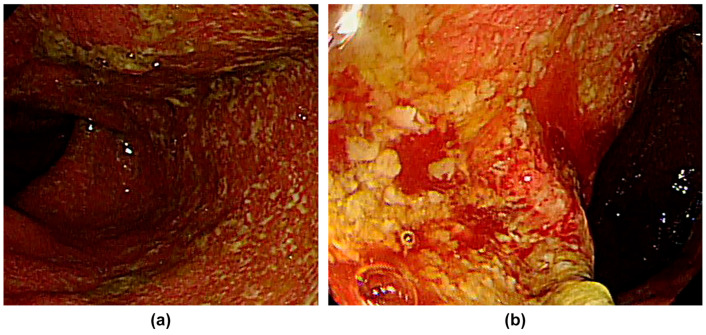
Colonoscopy before biological therapy. (**a**) Diffuse inflammation of the colonic mucosa, starting in the rectum and extending proximally; (**b**) Loss of vascularity to erosions, ulcers, and spontaneous bleeding.

**Figure 6:**
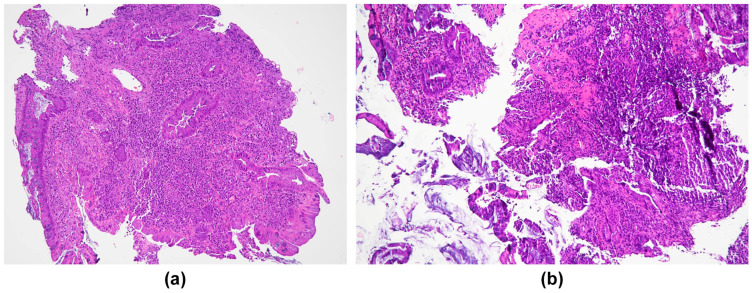
Pathology. Histopathological examination demonstrating focal architectural distortion, goblet cell depletion, cryptitis, and basal plasmacytosis with a Nancy index of 4, consistent with a diagnosis of ulcerative colitis; (**a**) Hematoxylin and eosin staining (original magnification, ×100); (**b**) Hematoxylin and eosin staining (original magnification, ×200).

**Figure 7:**
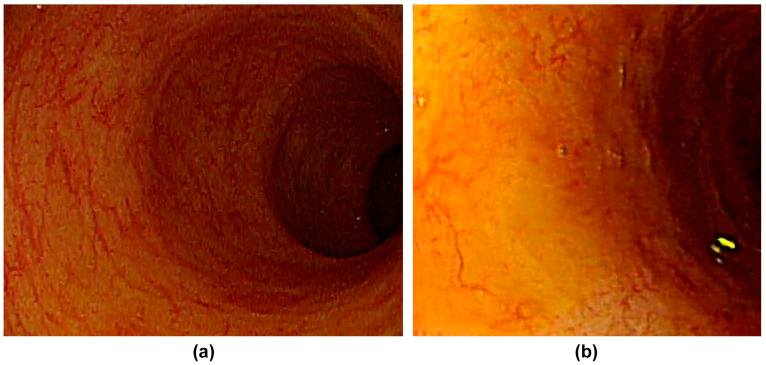
Colonoscopy after biological therapy. (**a**,**b**) Colonoscopic findings after vedolizumab therapy demonstrating near-complete endoscopic remission (Mayo endoscopic score 1).

**Table 1:** CARE Timeline of Clinical Course.

Time	Clinical Event
10 years before admission	Recurrent postprandial upper abdominal pain accompanied by intermittent fever.
At presentation	Jaundice, diarrhea (>5 times/day), hematochezia, weight loss (>10 kg), fatigue, and chronic pruritic rash.
Hospital admission	Laboratory tests demonstrated cholestatic liver injury and positive autoimmune markers.
Diagnostic work-up	ERCP and MRCP confirmed PSC. Autoimmune evaluation fulfilled the classification criteria for SLE. Colonoscopy and histopathology confirmed UC (Mayo endoscopic score 3, E3 disease extent).
Initial treatment	Intravenous corticosteroid 20 mg daily for 3 days, hydroxychloroquine 200 mg daily, antibiotics, and ursodeoxycholic acid.
After bleeding improvement	Oral prednisolone 10 mg daily and mesalazine 1000 mg daily were initiated.
Subsequent treatment	Azathioprine 50 mg every 3 days for 1 month.
Treatment modification	Azathioprine reduced to 25 mg every 3 days because of hepatotoxicity and combined with cyclosporine 50 mg daily.
Complication	Recurrent cholangitis with Escherichia coli bacteremia treated with flomoxef for 7 days. Follow-up blood cultures were negative.
Rescue therapy	Vedolizumab 300 mg administered at weeks 0, 2, and 6, followed by maintenance every 8 weeks.
Follow-up (1 year)	Clinical remission, resolution of hematochezia, improvement in inflammatory markers, and near-complete endoscopic remission (Mayo endoscopic score 1). PSC and SLE remained clinically stable. Patient remains on the liver transplantation waiting list.

**Table 2:** Clinical and Laboratory Outcomes Before and After Vedolizumab Therapy.

Parameter	Before Vedolizumab	After Vedolizumab (1-Year Follow-Up)
Stool frequency	5 times/day	1 time/day
Hematochezia	Present	Absent
Mayo score	11	1
Mayo endoscopic score	3	1
C-reactive protein	12 mg/L	1 mg/L
Erythrocyte sedimentation rate	17 mm/h	5 mm/h
Fecal calprotectin	256 μg/g	12 μg/g

## Data Availability

No new data were created or analyzed in this study.

## Abbreviations

The following abbreviations are used in this manuscript:

DIL	Drug-induced lupus
IBD	Inflammatory bowel disease
PSC	Primary sclerosing cholangitis
SLE	Systemic lupus erythematosus
